# Metabolic Cost of the Immune Response During Early Ontogeny of the Scallop *Argopecten purpuratus*

**DOI:** 10.3389/fphys.2021.718467

**Published:** 2021-09-01

**Authors:** Isis Rojas, Georgina A. Rivera-Ingraham, Claudia B. Cárcamo, Katherine Jeno, Erwin de la Fuente-Ortega, Paulina Schmitt, Katherina Brokordt

**Affiliations:** ^1^Doctorado en Acuicultura Programa Cooperativo Universidad de Chile, Universidad Católica del Norte, Pontificia Universidad Católica de Valparaíso, Coquimbo, Chile; ^2^Laboratorio de Fisiología Marina (FIGEMA), Departamento de Acuicultura, Facultad de Ciencias del Mar, Universidad Católica del Norte, Coquimbo, Chile; ^3^Laboratoire Environnement de Petit Saut, Hydreco-Guyane, Kourou, French Guiana; ^4^Centro de Innovación Acuícola (AquaPacífico), Universidad Católica del Norte, Coquimbo, Chile; ^5^Centro de Estudios Avanzados en Zonas Áridas (CEAZA), Coquimbo, Chile; ^6^Departamento de Ciencias Biomédicas, Facultad de Medicina, Universidad Católica del Norte, Coquimbo, Chile; ^7^Laboratorio de Genética e Inmunología Molecular, Facultad de Ciencias, Instituto de Biología, Pontificia Universidad Católica de Valparaíso, Valparaíso, Chile

**Keywords:** respiration rate, energy metabolism, enzymatic activity, mitochondria, bacterial infection, bivalve larvae

## Abstract

The scallop *Argopecten purpuratus* is an important resource for Chilean and Peruvian aquaculture. Seed availability from commercial hatcheries is critical due to recurrent massive mortalities associated with bacterial infections, especially during the veliger larval stage. The immune response plays a crucial role in counteracting the effects of such infections, but being energetically costly, it potentially competes with the physiological and morphological changes that occur during early development, which are equally expensive. Consequently, in this study, energy metabolism parameters at the individual and cellular levels, under routine-basal status and after the exposure to the pathogenic strain bacteria (*Vibrio splendidus* VPAP18), were evaluated during early ontogeny (trochophore, D-veliger, veliger, pediveliger, and early juveniles) of *A. purpuratus*. The parameters measured were as follows: (1) metabolic demand, determined as oxygen consumption rate and (2) ATP supplying capacity measured by key mitochondrial enzymes activities [citrate synthase (CS), electron transport system (ETS), and ETS/CS ratio, indicative of ATP supplying efficiency], mitochondrial membrane potential (ΔΨm), and mitochondrial density (*ρm*) using an *in vivo* image analysis. Data revealed that metabolic demand/capacity varies significantly throughout early development, with trochophores being the most efficient in terms of energy supplying capacity under basal conditions. ATP supplying efficiency decreased linearly with larval development, attaining its lowest level at the pediveliger stage, and increasing markedly in early juveniles. Veliger larvae at basal conditions were inefficient in terms of energy production vs. energy demand (with low *ρm*, ΔΨm, enzyme activities, and ETS:CS). Post-challenged results suggest that both trochophore and D-veliger would have the necessary energy to support the immune response. However, due to an immature immune system, the immunity of these stages would rely mainly on molecules of parental origin, as suggested by previous studies. On the other hand, post-challenged veliger maintained their metabolic demand but decreased their ATP supplying capacity, whereas pediveliger increased CS activity. Overall, results suggest that veliger larvae exhibit the lowest metabolic capacity to overcome a bacterial challenge, coinciding with previous works, showing a reduced capacity to express immune-related genes. This would result in a higher susceptibility to pathogen infection, potentially explaining the higher mortality rates occurring during *A. purpuratus* farming.

## Introduction

Early development is a sensitive and critical process in bivalve rearing and is thus often subject to large mortality rates (Nicolas et al., 1996; Balseiro et al., 2013). Massive mortalities in reared larvae and early juveniles have often been associated with the occurrence of bacterial infections (Dubert et al., 2017; Rojas et al., 2019). The immune response capacity plays a crucial role in counteracting the deleterious effects of such infections through the production of humoral immune-molecule (e.g., with recognition and microbicidal functions) and immune-cell (hemocytes) activities (e.g., chemotaxis, phagocytosis, and intracellular degradation of foreign material) [reviewed by Coyne (2011)]. These are energetically costly and potentially compete with the early developmental processes, which are equally expensive (Rodriguez et al., 1990; Garcia-Esquivel et al., 2001) due to the deep physiological and morphological changes they involve. These include, among others, shell formation, vellum loss, and all the necessary changes that allow the start of feeding through gill filtration (Bellolio et al., 1993). Therefore, a physiological tradeoff between these two processes may arise during early ontogeny. In order to evaluate this potential tradeoff, it is necessary to know the routine metabolic demands throughout early ontogeny and the cost of inducing the immune response in these stages.

Given that massive mortalities in hatcheries are recurrent during the larval period (Miranda et al., 2014), some studies have assessed the energetic metabolism in bivalve larvae, most commonly as the variation of energetic reserves (lipids, proteins, and carbohydrates; Whyte et al., 1987; Nevejan et al., 2003; Genard et al., 2011). In pectinids, studies addressing physiological changes during larval development are scarce, but some have focused on their effects on the energetic reserves, metabolic enzymes activity, and metabolic capacities (oxygen consumption rate) (MacDonald, 1988; Martínez et al., 1995). However, only a very few studies have evaluated the additional metabolic demands of counteracting pathogen infection during development. In oysters, it has been described that a bacterial infection triggering massive mortality events during larval development activates defenses and induces a decrease in certain parameters related to energy production, such as the activity of the mitochondrial enzyme citrate synthase (CS) (Genard et al., 2011).

The scallop *Argopecten purpuratus* is an important aquatic resource in the South American Pacific countries, especially for the Chilean and Peruvian aquaculture industries (FAO, 2018). The establishment and growth of this industry have been possible thanks to the production of early juveniles (seed) coming from either natural recruitment or from commercial farming. However, seed availability from the natural environment is still a critical point in the farming process, and the occurrence of disease outbreaks are increasingly frequent in commercial hatcheries (Merino et al., 2009), both together are a source of great economic loss (Riquelme et al., 1995; Rojas et al., 2015). Such massive mortalities most often occur during the veliger stage (Miranda et al., 2014), and have been related to infections caused mainly by the *Vibrio* bacteria (Garnier et al., 2007; Elston et al., 2008), namely *Vibrio splendidus* (Rojas et al., 2015, 2021), *V. bivalvicida* (Dubert et al., 2016; Rojas et al., 2019), and *V. alginolyticus* (Riquelme et al., 1996). Recently, the exposure of scallop larvae to a *V. splendidus* pathogenic strain (VPAP18) has been shown to induce an overexpression of immune-related genes covering different phases of immune response (sensing, signaling, and effectors), but only in late larval development stages of *A. purpuratus* (Rojas et al., 2021).

In the present study, two main questions were addressed: (i) Does the metabolic condition (associated with energy production and consumption) vary throughout early ontogeny in scallops? (ii) Are there differences in the metabolic energy demand between early stages of development when facing pathogenic bacteria? The latter could shed light on the metabolic cost of the immune response and the existence of a potential tradeoff between the energy demands inherent to each larval stage and their capability to fuel the immune response. To achieve these objectives, trochophore, D-veliger, veliger, pediveliger larvae, and early juveniles were considered both under “basal” conditions and in response to *V. splendidus* VPAP18 infection. An energy metabolic approach was used, which considered the following (1) metabolic demand/capacity at the organismic level, here the whole-animal oxygen consumption rate was measured, and (2) the cellular energy metabolism, with a purely mitochondrial focus. Mitochondria, being the main powerhouse in cells, provides an excellent view of the general energetic status. Thus, we measured CS activity (a key enzyme in the aerobic energy supply through the tricarboxylic acid cycle); electron transport system (ETS) activity (measure of the dehydrogenases and cytochromes that biochemically control cell respiration) (Maldonado et al., 2012); mitochondrial membrane potential (ΔΨm), an essential component in the process of energy storage during oxidative phosphorylation (Zorova et al., 2018); and mitochondrial density using *in vivo* imaging techniques. Studies on the energy metabolism during early ontogeny of commercially important bivalves and their metabolic capacity when facing a pathogenic bacterial challenge will shed light on the states that are energetically better or less prepared to cope with an infection (resistance/susceptibility), and thus optimize larvae rearing technologies or generate palliative strategies, for example through nutritional reinforcement.

## Materials and Methods

### Larvae Rearing

Larvae were obtained from an induced spawn as described in Rojas et al. (2021). Briefly, 80 mature adults (7.0 ± 0.5 cm in shell height) of *A. purpuratus* were collected from a culture at Tongoy Bay (Coquimbo region, Chile). Organisms were maintained in a 1,000-L aquarium with running filtered (1 μm) seawater for 2 days in the Central Laboratory for Marine Culture from the Universidad Católica del Norte at Coquimbo. Spawning was induced by exposing mature scallops to a high concentration of microalgae (*Isochrysis galbana* clone T-iso + *Chaetoceros calcitrans* + *Pavlova lutheri*, 17 × 10^6^ cell/ml). When the spawning began, the adults were separated to collect male and female gametes separately to avoid self-fertilization. Gametic products (oocytes from 65 individuals and sperms from another 15 individuals) were mixed in a ratio of seven to 10 sperms per oocyte, and the resulting eggs (~120 × 10^6^) were kept in a 250-L cylindrical tank. After 48 h, larvae were transferred to a recirculating aquaculture system (RAS) as modified from Merino et al. (2009). The RAS consisted of 12 cylinder–conical tanks of 200 L filled with filtered (1 μm) and sterilized (UV) seawater, maintained at room temperature (17 ± 1°C) and continuous aeration. Larvae were maintained in these tanks at a density of 30 larvae per ml. The 48-h postfecundation larvae (hpf) were daily fed with a microalgal mix containing *C. calcitrans* and *I. galbana* clone T-iso ensuring to maintain a concentration of 20,000 cells per ml in the tank. Feed rations were adjusted every 2 days. When larvae reached the metamorphosis stage, about 20 days postfecundation (dpf), cylindrical nets were submerged in the RAS tanks to allow settlement. Early juveniles were obtained 10 days post-settlement.

### Experimental Design and Bacterial Challenge

Half of the larvae used in this study were maintained undisturbed (for basal status assessments) whereas the other half were subjected to a bacterial challenge as in Rojas et al. (2021). The latter were exposed to the pathogenic strain of *Vibrio splendidus* (VPAP18), which was previously isolated from *A. purpuratus* larvae affected by a massive mortality event in a commercial hatchery in northern Chile (Rojas et al., 2015). This strain was cultivated in Tryptic Soy Broth (Difco) supplemented with 2% NaCl (Oxoid) medium at 22°C overnight in a mechanic shaker (100 rpm). The concentrated broth was diluted depending on the scallop larvae stage to infect. Trochophore (24 hpf) and D-veliger (48 hpf) stages were exposed to a bacterial dose of 90 colony forming units (cfu) per larvae. For veliger (8 dpf) and pediveliger (21 dpf) stages, 210 and 620 cfu per larvae were used, respectively. For early juvenile (31 dpf), a concentration of 3,500 cfu per individual was used. These were all sublethal infection doses, shown to be enough to trigger the immune response in *A. purpuratus*, based on the study from Rojas et al. (2021) and previous experiments, which considered the relative larval/juvenile volume and the larval/juvenile concentration per flask.

For every developmental stage, individuals were obtained from three randomly chosen tanks (out of the 12 set in place), pooled, and redistributed in experimental units for the bacterial challenge. The experimental units consisted of 1-L glass flasks. These were maintained, in all the cases, in a thermo-regulated bath at 17°C (hatchery rearing temperature), with a total of 16 units for each developmental stage and eight units per condition (undisturbed and challenged). Among these, six were used for the enzymatic analyses (i.e., CS and ETS) and the other two for *in vivo* measurements (oxygen consumption rate and confocal imaging analyses). Considering the timeline of the *V. splendidus* (VPAP18) infection course in *A. purpuratus* larvae (see Rojas et al., 2015), the experimental time for enzymatic analyses was 6 h, whereas for the rest of the analyses it was 2 h. The concentration of larvae per flask varied according to the developmental stage following the recommendations from Rojas et al. (2021), being 50,000 larvae/flask for trochophore and D-veliger stages, 35,000 larvae/flask for veliger, 25,000 larvae/flask for pediveliger, and 500 individuals/flask for early juveniles. In all the cases, the larvae were starved 24 h prior to the beginning of the experiment to avoid any possible influence on metabolic parameters. Once the experimental time was over, larvae were either used immediately for the *in vivo* determinations or concentrated by filtration (each flask represents one condition sample), centrifuged, and immediately frozen at −80°C for further (enzymatic) analyses.

### Oxygen Consumption

Respiration rate (RR) in each larval stage and early juveniles was measured in individual wells of a glass microtiter plate (Mikroglas Chemtech, Mainz, Germany) equipped with oxygen sensor spots (OXSP5, PyroScience GmbH, Aachen, Germany), as in Rivera-Ingraham et al. (2016a). The number of individuals in each well varied according to each developmental stage and was roughly equivalent to the farming densities aforementioned. Each well was filled with experimental seawater (sterile seawater for controls and sterile seawater + bacteria for challenged) to its maximum capacity (100 μl) and sealed with a coverslip ensuring the absence of air bubbles. All measurements were carried out using two four-channel fiber-optic oxygen meters (FireSting, PyroScience GmbH, Aachen, Germany) at controlled room temperature (17 ± 0.5°C). All measurements started at near air saturation (>98%). The O_2_ concentration was measured every 60 s using the PyroOxygen Logger Software until O_2_ was completely consumed in each well. Eight measurements were recorded simultaneously, with four replicas for each treatment. Once the measurements were taken, the number of larvae in each well was quantified using a Sedgewick-Rafter chamber for posterior standardization. Blank measurements were equally carried out using sterile seawater and sterile seawater with bacteria (in the absence of animals) to obtain the background and microbial respiration. RRs were calculated through linear regression by plotting air saturation as a function of time and corrected using the blank values. Only the values ≥70% of saturation were considered to avoid any possible (differential) influence of hypoxia on the animals. RR was expressed as individual (nmol O_2_·h^−1^·ind^−1^) and mass-specific (nmol O_2_·h^−1^·mg AFDW^−1^) rates. Ash-free dry weight (AFDW) was determined using the height vs. mass regression reported for the bay scallop *Argopecten irrandians* (Lu et al., 1999), except for trochophore for which this information was not available.

### Membrane Potential and Mitochondrial Density

The ρm and ΔΨm were quantified through *in vivo* confocal imaging after 2 h of bacterial exposure for every developmental stage, following the method explained by Rivera-Ingraham et al. (2016a). Briefly, in a cell-culture plate, undisturbed (control) and challenged larvae and early juveniles were aliquoted to be simultaneously stained with JC-10 and MitoTracker Deep Red 633 (MTK-DR) dyes. The JC-10 fluorophore (Enzo^®^ Life Sciences ENZ-5230), diluted in DMSO, was added to each aliquot to obtain a final concentration of 5 μM. JC-10 (ex: 517 nm) accumulates in the mitochondria in form of green monomers (em: 410–546 nm) in cases of low ΔΨm, but forms orange–fluorescent aggregates (em: 585–700 nm) at higher ΔΨm. ΔΨm was calculated as the fluorescence intensity ratio between both green and orange emission channels. The MitoTracker Deep Red 633 (MTK-DR, Molecular Probes M-22426) fluorophore (ex: 644; em: 645–700 nm), diluted in DMSO, was also added for ρm estimation, given that this molecule becomes fluorescent once it accumulates in the lipid environment of mitochondria. Both fluorophores were simultaneously incubated in darkness for 30 min. About 10 min before the imaging analysis, animals were anesthetized with a solution of 7.16% MgCl_2_ ·6H_2_O in seawater (Pfannkuche and Thiel, 1988).

Samples were observed in a Zeiss LSM 800 confocal microscope (Carl Zeiss, Heidelberg, Germany) equipped with diode lasers. Visualization and imaging were carried out using a Plan-Neofluar 40×/1.3 Imm Korr objective. To ensure that we would, in all the cases, focus on the same Z range, the area of interest was established to be the mantle edge, in which the preliminary observations demonstrated to have the highest mitochondrial density. A minimum of five individuals were considered for every developmental stage and every condition (undisturbed and bacterial exposed). For each animal analyzed, this region was located using transmission light to avoid photobleaching. Then, three single pictures (512 × 512 pixels) were taken for JC-10 monomers, JC-10 aggregates, and MTK-DR, respectively. Pictures were taken in this order to reduce the impact of phototoxicity in the measurement of the most sensitive parameter measured (here ΔΨm).

The fluorescence was measured only in the mantle edge of each larva or juvenile considered, and in areas of highest, albeit not saturated, MTK-DR fluorescence. For each sample (individual larva), 10 regions of interest (ROIs) were considered. Every ROI consisted of a square of an approximate area of 0.5 μm^2^. For each ROI, three values were calculated: (a) the average fluorescence of MTK-DR, (b) the average orange fluorescence for JC-10 aggregates, and (c) the average green fluorescence of JC-10 monomers. The ρm for each larva was calculated as the average value of all 10 ROIs. The ΔΨm of a given ROI was calculated as the ratio between JC-10 aggregates and monomers, and the average of the 10 ROIs was considered as the value for a given sample. Then, the average ρm and ΔΨm for each developmental stage and treatment was calculated. Fluorescence quantifications were made using the software FIJI-ImageJ2 (Schindelin et al., 2012; Rueden et al., 2017) and the Bio-Formats plugin (Linkert et al., 2010).

### CS and ETS Activity

Samples were weighted and homogenized on ice in a buffer containing 0.1% Tween 20, 2mM EDTA-Na_2_, 5mM EGTA, 150mM KCl, 1mM dithiothreitol, and 50 mM imidazole-HCl in a proportion of 1:5 W:V. The homogenates were centrifuged at 600 *g* for 10 min at 4°C, and the supernatant was immediately used for enzymatic assays.

An aliquot of the supernatant was used for determining CS activity, following the methodology described by Brokordt et al. (2000) for *A. purpuratus*. Briefly, the supernatant was diluted at a 1:3 ratio (V:V) in a reaction mixture containing 75 mmol·L^−1^ TRIS-HCl, 0.3 mmol·L^−1^ oxaloacetate, 0.1 mmol·L^−1^ DTNB (5,5-dithio-bis-2-nitrobenzoic acid), and 0.2 mmol·L^−1^ acetyl CoA. The enzymatic activity was measured using a spectrophotometer EPOCH (Biotek) at 412 nm (absorbance) at controlled room temperature (17°C). DTNB molar extinction used was 13.6 mM^−1^·cm^−1^. All assays were run in duplicate, and the specific activities were expressed in international units (IU) per mg of the wet mass.

The rest of the supernatant was used for determining the ETS activity, using the method proposed by Packard (1971) with minor modifications. This method estimates the maximum potential activity of the electron transporters in the respiratory chain at the mitochondrial level (Saavedra et al., 2016). An aliquot of the supernatant was diluted at a 1:10 ratio (V:V) in a buffer containing 7 mM TRIS, 5% polyvinylpyrrolidione (PVP), 153mM MgSO_4_, and 0.1% Tween 20. Enzyme activity was determined in a spectrophotometer EPOCH (Biotek) using a reaction mixture containing 75mM TRIS, 0.1% Tween 20, 1.7mM NADH, 250 μM NADPH, and 0.2% iodonitrotetrazolium (INT). Absorbance changes were measured at 490 nm. The molar extinction of INT used was 15.9 mM^−1^·cm^−1^. ETS activity was expressed in IU per mg of wet mass. In addition, ETS was also standardized by CS activity as a proxy of ATP production efficiency.

Results are presented as means ± standard errors of the mean (SE). Shapiro–Wilk's test was used to test normality whereas homoscedasticity was tested with the Fligner–Kileen test. Results meeting the requirements for parametric analyses (i.e., RR, CS, ΔΨm, and ρm) were evaluated by one-way ANOVA to evaluate differences among developmental stages at routine/basal levels. A two-way ANOVA was applied to compare the effect of the bacterial challenges on every parameter assessed among developmental stages. The ETS activity assumptions for which parametric analyses were not met, a non-parametric Kruskal–Wallis test was applied. In the case of ETS:CS ratio, a robust one-way ANOVA was applied (Mair and Wilcox, 2020). Tests were considered statistically different with *P* < 0.05. All statistical analyses were conducted using R version 3.6.1 (R Core Team, 2013) with the “agricolae” package (de Mendiburu and Yaseen, 2020). A robust test was made using the “WRS2” package (Mair and Wilcox, 2020).

## Results

### Oxygen Consumption

**Figure 1:**
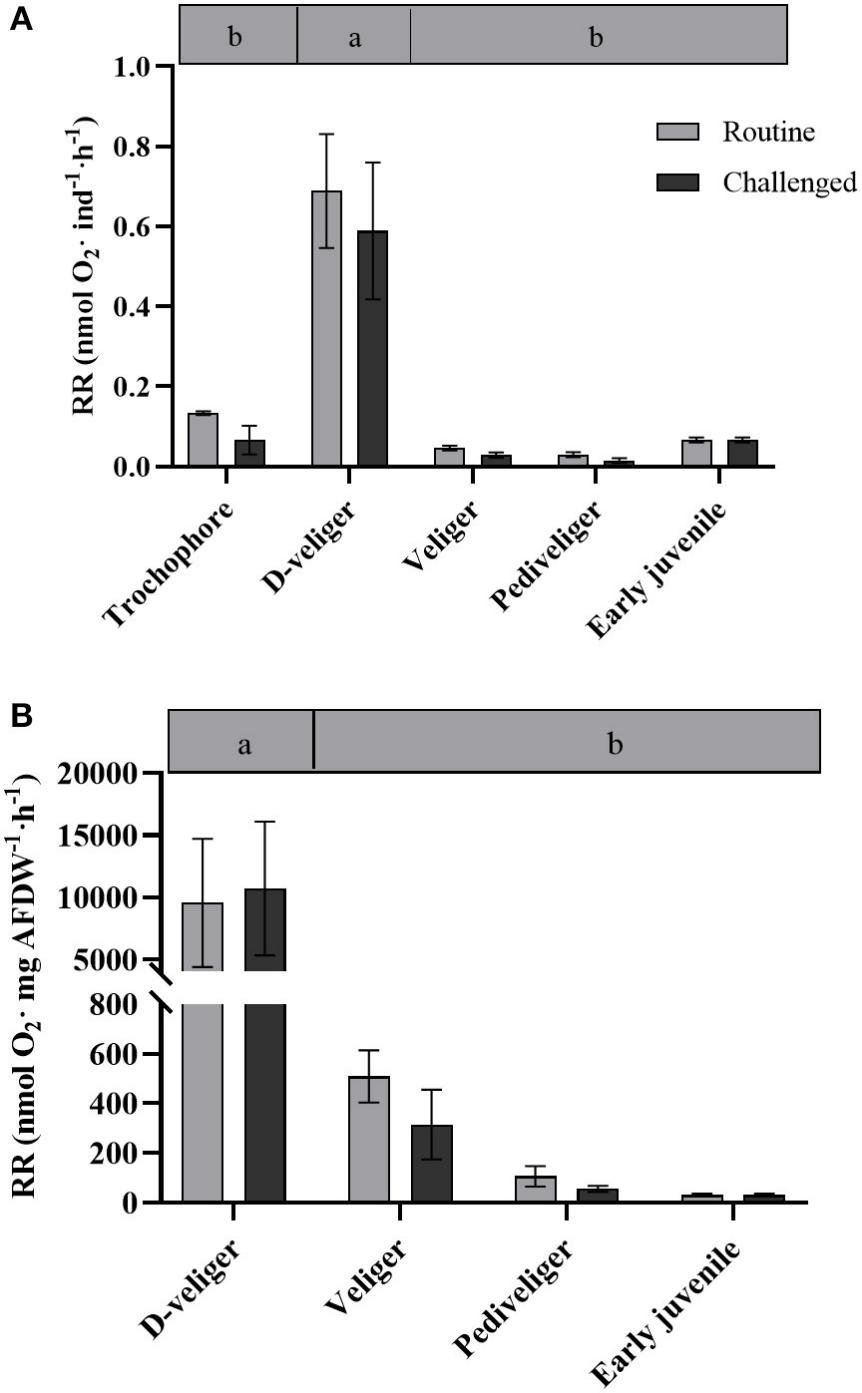
**(A)** Respiration rate per individual (nmol O_2_ ind^−1^· h^−1^) and **(B)** per mass (nmol O_2_·mg AFDW·h^−1^) throughout larval development and in early juveniles from the scallop *Argopecten purpuratus*. Different lowercase letters inside bars at the top of the graph indicate significant differences among developmental stages in basal conditions (*P* < 0.05). *n* = 4 pools per condition.

Total and mass-specific RRs under routine conditions varied significantly among larval stages (Table 1), with values up to six to 20 times higher in D-larvae stage than in the other developmental stages (Figure 1). The factorial analysis (two-way ANOVA) showed that RRs were affected only by the developmental stage, and not by the bacterial challenge or the interaction between these factors (Table 2).

**Table 1:** One-Way ANOVA evaluating the effect of the developmental stage under basal conditions on respiration rates (RR), mitochondrial density (ρm), mitochondrial membrane potential (ΔΨm), citrate synthase activity (CS), electron transport chain activity (ETS), and ETS:CS ratio (robust ANOVA) throughout larval development and in early juveniles from the scallop *Argopecten purpuratus*.

**Parameter**	**Source**	***Df***	**SS**	**MS**	***F***	***P***
RR (per individual)	Stage	4	0.5398	0.1349	10.78	**0.0004**
	Residuals	13	0.1627	0.0125		
RR (mass-specific)	Stage	3	103,93	34,645	12.9	**0.0008**
	Residuals	10	26,856	2,686		
ρm	Stage	4	1.04 × 10^−10^	2.61 × 10^−09^	154.6	**<** **0.0001**
	Residuals	14	2.36 × 10^−08^	1.69 × 10^−07^		
ΔΨm	Stage	4	297.03	74.26	13.32	**0.0001**
	Residuals	14	78.05	5.57		
CS activity	Stage	4	7,681	1,920	70.82	**<** **0.0001**
	Residuals	54	1,464	27.1		
ETS activity	Stage	4	7,369	1,842	14.15	**<** **0.0001**
	Residuals	22	2,865	130.2		

*The analyses were conducted on raw data. Statistically significant results (P < 0.05) are indicated in bold*.

**Table 2:** Two-Way ANOVAs evaluating the effect of the developmental stage and the bacterial challenge on respiration rates (RR), mitochondrial density (ρm), mitochondrial membrane potential (ΔΨm), citrate synthase activity (CS), and ETS:CS ratio throughout larval development and in early juveniles from the scallop *Argopecten purpuratus*.

**Parameter**	**Source**	***Df***	**SS**	**MS**	***F***	***P***
RR (per individual)	Stage (S)	4	1.2647	0.3161	23.925	**<** **0.0001**
	Challenge (C)	1	0.0001	0.0001	0.008	0.93
	S × C	4	0.0069	0.0017	0.13	0.97
	Residuals	26	0.3436	0.0132		
RR (mass-specific)	Stage	3	23.268	7.756	262.764	**<** **0.0001**
	Challenge	1	0.078	0.078	2.63	0.121
	S × C	3	0.144	0.048	1.622	0.216
	Residuals	20	0.59	0.03		
ρm	Stage	4	1.54 × 10^10^	3.84 × 10^9^	250.61	**<** **0.0001**
	Challenge	1	2.14 × 10^8^	2.14 × 10^8^	13.95	**0.0008**
	S × C	4	2.98 × 10^9^	7.46 × 10^8^	48.64	**<** **0.0001**
	Residuals	29	4.45 × 10^8^	1.53 × 10^7^		
ΔΨm	Stage	4	781.7	195.42	35.275	**<** **0.0001**
	Challenge	1	0.1	0.08	0.014	0.905
	S × C	4	167.6	41.9	7.563	**0.0002**
	Residuals	32	177.3	5.54		
CS activity	Stage	4	7681	1920.1	94.294	**<** **0.0001**
	Challenge	1	17	16.8	0.825	0.368
	S × C	4	450	112.4	5.519	**0.0009**
	Residuals	49	998	20.4		
ETS:CS	Stage	4	0.5207	0.1301	26.893	**<** **0.0001**
	Challenge	1	0.0008	0.0007	0.157	0.694
	S × C	4	0.0366	0.0091	1.891	0.127
	Residuals	48	0.2323	0.0048		

*The analyses were conducted on raw data. Statistically significant results (P < 0.05) are indicated in bold*.

### Mitochondrial Density and Membrane Potential

**Figure 2:**
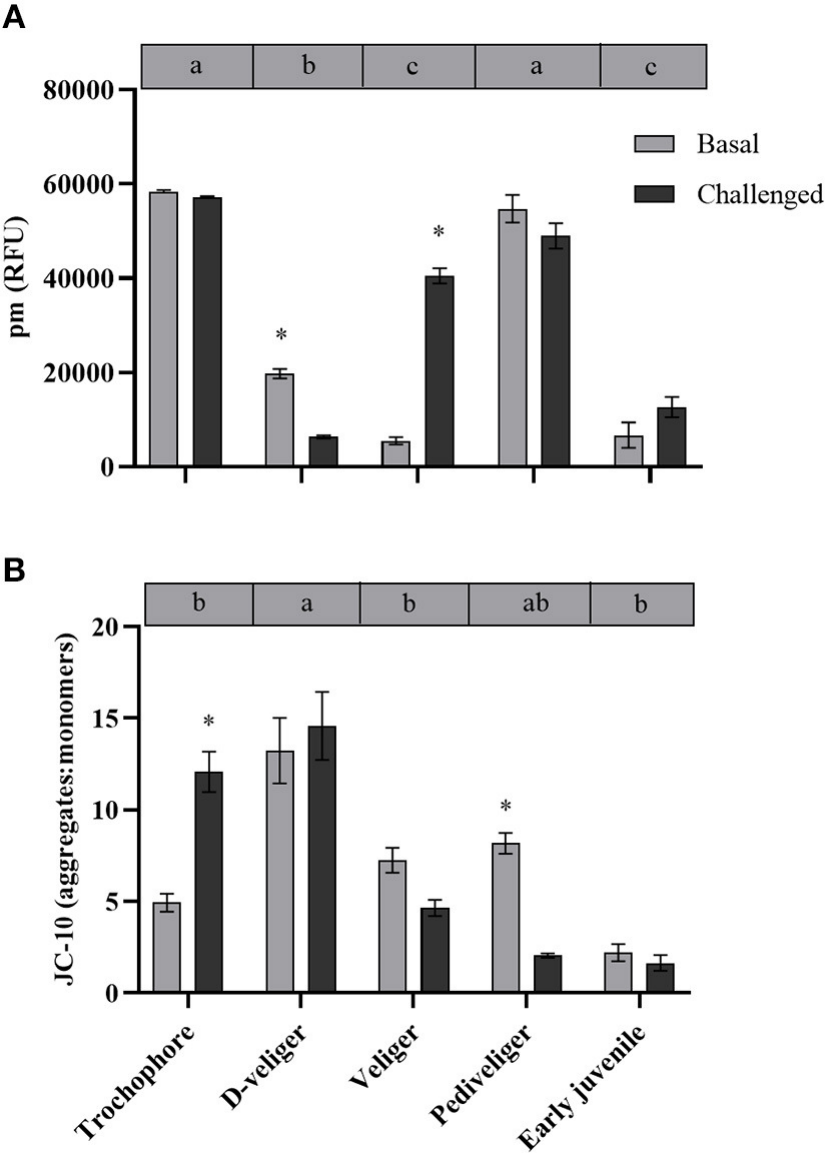
**(A)** Mitochondrial density (ρm) and **(B)** mitochondrial membrane potential (ΔΨm) throughout larval developmental stages and in early juveniles from the scallop *Argopecten purpuratus*. Here, ρm is expressed as relative fluorescence units (RFU) while ΔΨm is expressed as a JC-10 fluorescence intensity ratio (aggregate/red: monomer/green). Different lowercase letters inside bars at the top of each graph indicate significant differences among developmental stages in basal conditions (*P* < 0.05). Asterisks indicate significant differences between control and challenged groups (*P* < 0.05). *n* = 4 pools per condition.

**Figure 3:**
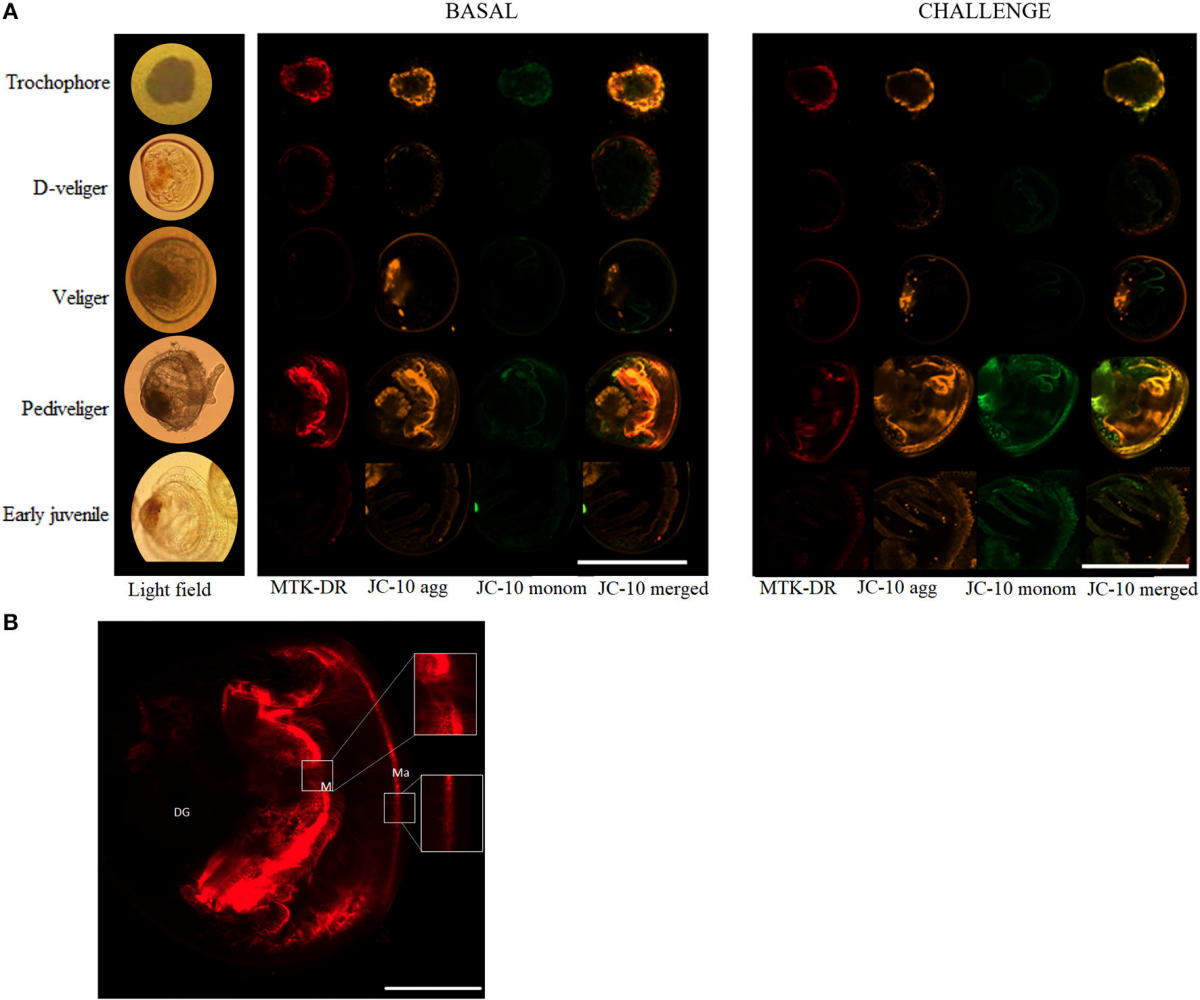
**(A)** Representative images obtained from *in vivo* analysis of the mitochondrial relative density (ρm) and mitochondrial membrane potential (ΔΨm) throughout larval development and in early juveniles of the scallop *Argopecten purpuratus* in basal (left) and *Vibrio* challenged (right) conditions. Here ρm is stained with MTK-DR dye marked in red while ΔΨm is stained with JC10 dye, monomers are marked in orange and green, the rightmost column is the image resulting from merging JC-10 orange and green channels. Scale bar measure is 160μm. **(B)** Details of the representative image obtained from *in vivo* analysis of the ρm in pediveliger undisturbed larvae. Scale bar measure is 40μm. Ma, Mantle; M, Mouth; DG, Digestive gland.

*In vivo* analysis of ρm in the edge of the mantle showed a significant variation throughout the early developmental stages of *A. purpuratus* under basal conditions (Table 1, Figures 2A, 3). Compared to the other larval stages, veliger larvae exhibited the lowest ρm at basal levels, followed by early juveniles. Trochophores and pediveligers showed the highest levels (Figures 2A, 3). Thus, under basal conditions, ρm across larval stages followed a clear U-shape pattern to later decrease in early juveniles. A cross effect between developmental stage and bacterial exposure was also found (Table 2). Interestingly, only veliger larvae increased their ρm as a response to the bacterial challenge, increasing by almost 8-fold. Early juveniles showed a similar tendency, although values did not reach statistical significance. Oppositely, the same bacterial challenge caused a 2-fold decrease in D-larvae ρm. The ΔΨm (JC-10 fluorescence ratio), also varied significantly along early development (Table 1, Figures 2B, 3), showing the highest basal values in D-larvae and pediveligers and the lowest in trochophores, veliger, and early juveniles. Statistically, a cross effect between developmental stage and bacterial challenge was found (Table 2). When exposed to a bacterial challenge, trochophore larvae increased their ΔΨm by almost three times. On the contrary, the same challenge caused pediveliger larvae to decrease their ΔΨm by the same amount (Figure 2B). The ΔΨm tended to decrease in veliger larvae exposed to the vibrio pathogen. D-veliger larvae and early juveniles did not vary their ΔΨm in response to the bacterial challenge, maintaining the high level observed under basal conditions in the case of D-veliger.

### CS Activity

**Figure 4:**
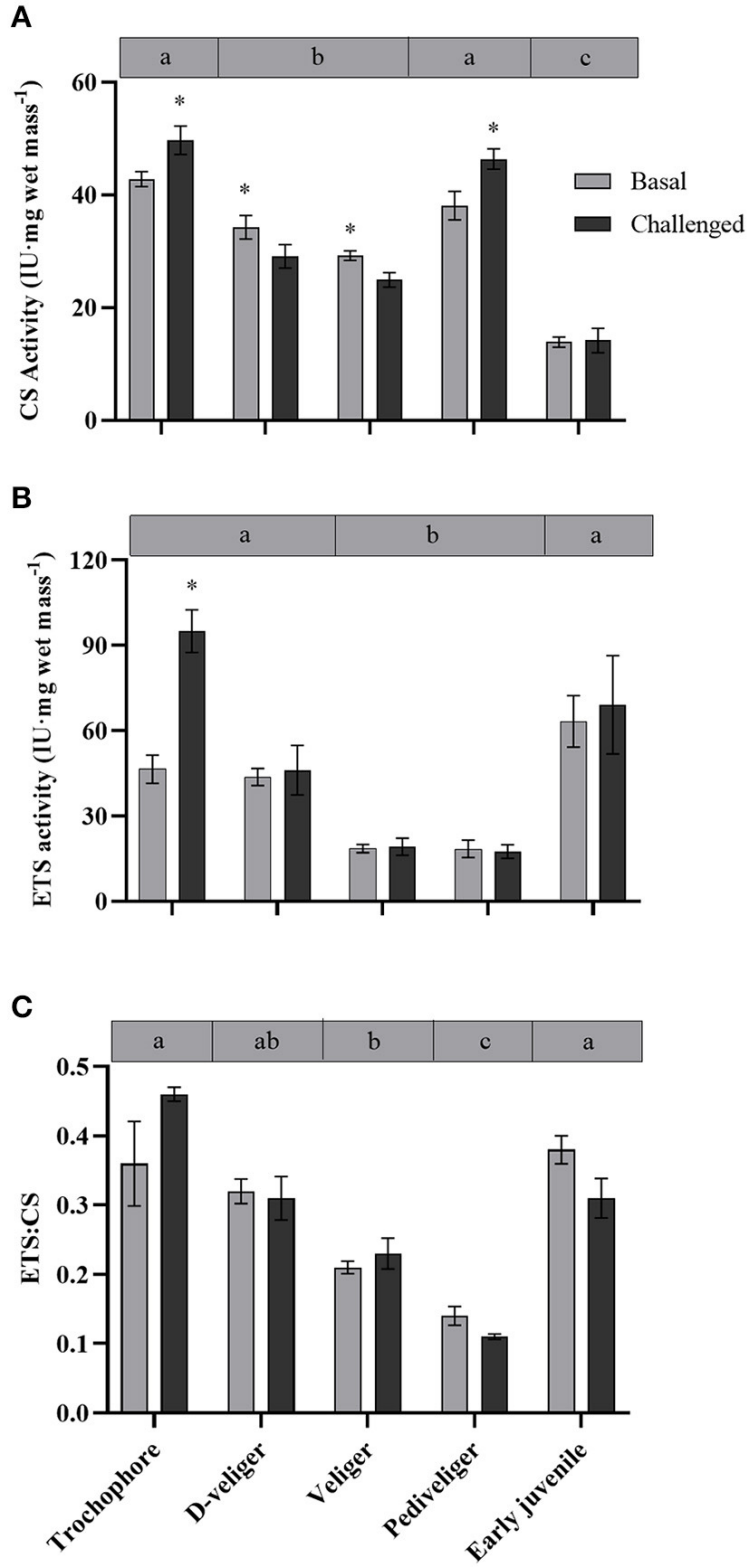
**(A)** CS, **(B)** ETS activity, and **(C)** ETS:CS ratio throughout larval development and in early juveniles from the scallop *Argopecten purpuratus*. Different lowercase letters inside bars at the top of the graph indicate significant differences among basal developmental stages (*P* < 0.05). Asterisks indicate significant differences between control and challenged groups (*P* < 0.05). *n* = 6 per condition.

Citrate synthase activity varied during the early ontogeny of the scallop *A. purpuratus*, showing a “U” shape pattern during larval development, and later decreasing in the early juveniles (Table 1, Figure 4A). Under basal conditions, the lowest CS activity among larval stages was shown by both D-veliger and veliger, respectively, but early juveniles presented lower CS activities than any of the larval stages. A significant cross effect between developmental stage and bacterial exposure was found (Table 2). In trochophore and pediveliger stages, CS activity increased in response to the bacterial exposure; conversely, the same challenge decreased CS activities during the D-veliger and veliger stages.

### ETS Activity and Energy Production Efficiency

Electron transport system activity varied throughout the early ontogeny of the scallop *A. purpuratus* (Table 1, Figure 4B). Trochophore, D-veliger, and early juveniles presented the greatest ETS values. Contrarily, veliger and pediveliger larvae showed the lowest ETS activities, these being on average 50% lower than the other stages. *Vibrio* exposure caused a significant increase in the ETS activity in trochophore larvae, but it did not affect the values of any of the other developmental stages (*P* = 7.86 × 10^−6^, *Df* = 9, X^2^ = 39.19, Figure 4B).

The ETS:CS ratio (Figure 4C) was here used as an indication of ATP supplying efficiency by mitochondria. The analysis showed that this ratio varied among larval stages and in the early juveniles [*F*_(4, 25.4)_ = 19.22, *P* = 0.000, Explanatory measure of effect size: 0.7]. In this regard, the results showed that at basal levels, both veliger and pediveliger larvae presented the lowest energetic activity compared with the other larval stages and early juveniles. No statistically significant effect of the bacterial exposure was found (Table 2).

## Discussion

The ability to initiate defense against pathogens in bivalve early ontogeny is highly dependent on the adequate energy allocation between immune response and other critical processes like those associated with development (Bassim et al., 2014). Considering this, and the economic losses caused by *Vibrio* outbreaks, we investigated from an organism and cellular perspective development-associated metabolic changes and how infection by the pathogenic strain of *V. splendidus* (VPAP18) affects the energy metabolism (energy demand and energy supplying efficiency) throughout early ontogeny of the scallop *A. purpuratus*. Overall, our results showed that both the morphological changes associated with the development and pathogen exposure affected the energy metabolism of this scallop, with more persistent effects on the veliger larval stage. Interestingly, it is at this larval stage where massive mortalities have been more often reported for *A. purpuratus* (Miranda et al., 2014).

### Metabolic Changes Through Early Ontogeny of Scallops

Although several studies have described the metabolic capacities and metabolic demands in adult scallops (Wang et al., 2012; Brokordt et al., 2015, 2019), such information is scarce for early developmental stages (larvae and juveniles). In the late 1980s and 90s, some studies described certain energetic aspects of scallop larvae, like filtration rate (energy uptake) and biochemical changes (MacDonald, 1988; Farías et al., 1998). However, energy metabolism parameters at the individual and cellular levels at such early stages have rarely been addressed (Wang and Zhang, 1995; Lu et al., 1999). In the present study, whole-animal metabolic demand was measured *via* RR under undisturbed (routine) conditions. Results indicate that D-veliger larvae consume eight to 20-fold (absolute and relative rates, respectively), more oxygen than any other larval stages analyzed. This may be because D-larvae, compared with more advanced stages, tend to swim continuously, beating cilia and clapping their newly formed valves. In this regard, it is reported that a great portion of metabolic energy is lost by larval activity [Reviewed by Sprung (1984)]. Both the swimming habits and the activity of the larvae mainly affected oxygen consumption rates, as was previously described for *Mytilus edulis* larvae (Sprung, 1984). The routine oxygen consumption rate reported for veliger *A. irradians* larvae (0.00135 ± 0.00052 μL O_2_ h^−1^·ind^−1^) is similar to what was observed in this study. In the Antarctic sea urchin, basal metabolic rates followed a similar pattern to the one described here (Marsh et al., 1999), where RRs started low, reached a maximum in pluteus larval stage, and then decreased during the later developmental stages, being determined not only by cell numbers but also by changes in specific biochemical activities. In older larvae, size-specific RRs may be lower due to the larger presence of different cell types presenting low metabolic activities (Marsh et al., 1999). It should also be noted, however, that during the D-veliger stage, larvae initiate all the morpho-anatomical changes for the development of their digestive system (Bellolio et al., 1993), all of which are energy-consuming processes. Furthermore, the fact that D-veliger larvae start relying on exogenous nutrition implies the beginning of a larger exposure and exchange with the external environment, and with all the interactions with potential pathogens that imply and require the setup of the (also energy-hungry) immune system, altogether explain the high RR recorded for this larval stage.

Aerobic eukaryotes synthesize ATP mainly by the oxidative phosphorylation (OxPhos) on the inner mitochondrial membrane, where the ΔΨm is the main proton motive force used by ATP synthase to produce ATP (see review by Kadenbach, 2003). Due to the profound effects, it has on ATP synthase activity, ΔΨm is thus strongly controlled *in vivo*. Under undisturbed (near-basal) conditions ΔΨm is generally maintained at low levels to stimulate the activity of OxPhos proton pumps and thus mitochondrial respiration, whereas higher levels have an inhibitory effect (reviewed by Kadenbach, 2003). Throughout *A. purpuratus* development, the basal ΔΨm showed a similar pattern to whole-animal (and the estimated relative) oxygen consumption, which may be indicative that the potential energy production capacity of mitochondria is proportional to the energy requirements during larval development. The ATP/ADP ratio is indeed known to control ΔΨm, with lower values (indicative of energy expense) stimulating the ATP synthase and respiration while resulting in decreased ΔΨm (Nicholls and Ferguson, 2002). But this control mechanism has been suggested to be switched off under stress conditions, causing a strong ΔΨm increase to maintain maximal rates of ATP synthesis during phases of high rates of ATP utilization and as a consequence of calcium-activated dephosphorylation of the cytochrome *c* oxidase (Robb-Gaspers et al., 1998a,b). This could explain the high basal ΔΨm recorded for D-veliger larvae, which was accompanied by the high RR, suggestive that during this stage, larvae are under stressful conditions with the beginning of the exogenous nutrition and the first deep interactions with the external environment. Although mitochondrial density was low at this larval stage, these would be efficient in their ATP supplying capacity, as indicated by the high ETS activity and ETS/CS ratio, in comparison with the other stages. In veliger larvae, the subsequent stage, absolute RR significantly decreased, but in terms of mass-specific RR, it was 5- and 20-fold higher than the pediveliger and juvenile RR, respectively. However, no differences were observed for ΔΨm among these stages. Also, the ETS activity and ETS/CS ratio were low for the veliger larvae, which in addition to the lowest mitochondrial density, are suggestive of a reduced energetic supplying capacity during this development stage.

The aerobic capacity of energy production, measured as the CS enzyme activity (key in regulating the TCA cycle), at the basal level, showed a “U” shape pattern throughout the larval development of *A. purpuratus*, with trochophore and pediveliger stages showing the greater CS activity. A similar pattern was previously described for *Drosophila melanogaster* by Merkey et al. (2011), who explained that this may be due to changes in the quantity of potentially active aerobic tissue. Also consistent with our results, previous works on oysters show that CS activity is lower in the D-veliger stage, reaches its maximal levels during the pre-metamorphic stage, and later decreases in early juveniles (Genard et al., 2011). CS activity values are often used as a proxy for mitochondrial number or density (ρm), hence confirming the live-imaging results with MTK-DR at whole-organism (larvae) level, which followed the same U shape pattern. It is however interesting to highlight that the highest ρm values in trochophore and pediveliger larvae were located at the mantle edge while this pattern was not observed in the other larval stages or juveniles. This is to be expected if we consider that trochophore and pediveliger larvae present an area with abundant cilia (Bellolio et al., 1993) and that motile structures, like any other energy-consuming organelle, are associated with cuffs of mitochondria that ensure energy supply (Bereiter-Hahn and Vöth, 1994). This was for example reported by Rivera-Ingraham et al. (2016b), who found densely packed mitochondria associated with the cilial basal bodies of the ciliated epidermal cells compositing *Mytilus edulis* gills filaments. In this regard, MTK-DR live images show strong staining at the entrance of the mouth, indicating another area of full-packaged mitochondria in pediveliger larvae.

Overall, among the evaluated development stages of *A. purpuratus*, trochophore larvae were apparently the most efficient in terms of energy-supplying capacity under basal conditions, both at the TCA cycle, ETS activity, and ETS:CS ratio. This could be due to multiple biological processes such as cellular and metabolic processes, biological regulation, and organization and biogenesis that must be fulfilled in this larval stage, as has been demonstrated at the transcriptomic level in early larvae of the mussel *M. edulis* (Bassim et al., 2014). Interestingly, ATP supplying efficiency by mitochondria as indicated by ETS:CS ratio decreased linearly with larval development, attaining the lowest level at the premetamorphic pediveliger stage, and increasing markedly in early juveniles.

Altogether, all these data reveal that metabolic demand and metabolic capacity vary significantly throughout *A. purpuratus* early development. Herein, we evidence that veliger larvae compared with other developmental stages, are inefficient in terms of energy production vs. energy demand, as confirmed by the low mitochondrial density and low ΔΨm, along with fewer activity of key mitochondrial enzymes and ETS:CS ratio.

### Metabolic Cost of Immune Response During Early Development

Immune response is an energetically expensive process because it implies the recruitment and activity of specialized cells (namely hemocytes in mollusks), and the production and action of specific molecules involved in sensing and opsonizing foreign particles as well as in antimicrobial functions. Although in adult scallops the immune response has been extensively investigated at both the cellular and humoral levels (Pérez et al., 2016; González et al., 2017; Brokordt et al., 2019), only few studies have addressed the immune response (using the expression of immune genes) in the larval phase (Yue et al., 2013a; Rojas et al., 2021) and one in early juveniles (postsettlement) (Rojas et al., 2021). These studies were, respectively, carried out in *Chlamys farreri* and *A. purpuratus* showing how immune responses are indeed developed since early ontogeny, although the induction of immune-related genes occurs later (pediveliger and early juvenile) in *A. purpuratus* than in *C. farrerri* (D-veliger and veliger). The present study constitutes, to the knowledge of the authors, the first evaluation of the metabolic cost induced by the exposure of a pathogenic bacterium in the different stages of the early development of *A. purpuratus*.

Respiration rates did not vary in the course of the first hours of bacterial exposure and followed the pattern of undisturbed larvae and early juveniles. Therefore, independent of the bacterial challenge, D-veliger maintained the highest RR (at individual and mass-specific levels), followed by veliger, pediveliger, and juveniles (at mass-specific levels). However, a clear and consistent induction of ATP supply was observed, but mainly in the postchallenged trochophore, as indicated by an increment of ΔΨm (by over 2-fold), CS activity, and ETS enzymes (and a tendency in ETS:CS ratio). These responses are expected under stress conditions (here a pathogen exposure), aiming to produce the energy required to fuel the necessary pathways to counteract the deleterious effects of such stresses (Kadenbach 2003). Interestingly, although post-challenged D-veliger maintained the highest energy demand (RR), mitochondrial density (as indicated by live imaging and CS activity) decreased markedly, but ΔΨm and ATP supplying efficiency (ETS:CS ratio) were as high as under the basal levels. These results suggest that both, trochophore and D-veliger would have the necessary energy to support immune response. On the other hand, post-challenged veliger maintained their RR, increased mitochondrial density, but decreased their CS activity, showing a tendency to decrease their ΔΨm and maintain a low ETS:CS ratio. Post-challenged pediveligers maintained the highest ρm, decreased ΔΨm markedly, and maintained the lowest ETS:CS ratio, but increased CS activity. These results suggest that both veliger and pediveliger (to a lesser extent) stages would have a limited energy supply to support immune responses. In general, early juveniles followed a distinct pattern, showing that the lowest RR that coincides with the lowest mitochondrial density (as indicated by live-imaging and CS activity) and ΔΨm, but one of the highest OxPhos rates and ATP-supplying efficiencies, which were maintained even after the bacterial exposure. These results suggest that early juveniles are a less energy-demanding stage at the basal level, are less perturbed by the presence of the bacteria, and would have a good capacity to energetically fuel the immune response.

The present findings are partially consistent with the expression pattern of immune genes observed in the same developmental stages of *A. purpuratus* (Rojas et al., 2021). This previous study showed that early juveniles overexpress a Toll-like receptor (*ApTLR*) in response to bacterial challenge. The latter also showed a similar overexpression of two antimicrobial effectors, a ferritin (*Apfer1*) and a big defensin antimicrobial peptide (*ApBD1*) in pediveliger and early juveniles. However, trochophore and D-veliger, which appear to be better prepared to support immune response in terms of energy supply, did not overexpress immune genes after *Vibrio* exposure (Rojas et al., 2021). Thus, the immune response would be likely limited by the level of maturation of the immune system in these early stages. However, parental transfer of some immune effectors has been suggested to occur, acting as protection during these early stages (Yue et al., 2013b; Jia et al., 2017; Rojas et al., 2021), but which would not be present in veliger larvae (Jia et al., 2017). It has been indeed suggested in the Mediterranean mussel (*M. galloprovincialis*) that veliger larvae have fewer differentially expressed genes than other larval stages, which would be associated with a lower energy expenditure for transcription and subsequent protein synthesis (Moreira et al., 2018). Moreover, present results suggest that the lack of induction of immune molecules after pathogen exposure might be limited by a low capacity to supply the necessary energy. This would potentially explain why pathogenic outbreaks occur (or affect) more often during the veliger larval stage (Miranda et al., 2014). We thus suggest that a potential energetic compromise between the morpho-anatomical changes and the immune response capacity exists during this larval stage, but a more in-depth study would be necessary to elucidate this tradeoff.

### Conclusion

The traditional farming methods of the scallop *A. purpuratus* involves the daily handling of the larvae and their confinement in small volumes while the tank cleaning tasks are carried out, which generates a potential handling stress in the larvae (Pérez et al., 2016) and could increase their susceptibility to pathogen attack. This point is especially relevant now that all evidence collected in the frame of the present study allows us to infer that the veliger stage would have lower energy efficiency along with the incapacity to express immune-related genes (Rojas et al., 2021). This would explain why this larval stage shows the highest mortality rates in commercial crops (i.e., between 5 and 7 days of farming). With these results, we propose that technical improvements in farming, that is, considering engineering or administration of immunomodulators, should aim to overpass this critical point aiming to improve the energy condition of the veliger larvae so that they may achieve a higher settlement index and a lower vulnerability to pathogen infections.

## Data Availability Statement

The raw data supporting the conclusions of this article will be made available by the authors upon request.

## Author Contributions

IR: methodology, validation, investigation, visualization, data curation, formal analysis, writing the original draft, reviewing, and editing. GR-I: methodology, validation, investigation, visualization, resources, reviewing, and editing. CC: software, reviewing and editing, project administration, and funding acquisition. KJ: investigation, visualization, reviewing, and editing. EF-O: resources, reviewing, and editing. PS: conceptualization, reviewing and editing, and funding acquisition. KB: conceptualization, methodology, investigation, supervision, resources, writing the original draft, reviewing, editing, project administration, and funding acquisition. All authors contributed to the article and approved the submitted version.

## Conflict of Interest

The authors declare that the research was conducted in the absence of any commercial or financial relationships that could be construed as a potential conflict of interest.

## Publisher's Note

All claims expressed in this article are solely those of the authors and do not necessarily represent those of their affiliated organizations, or those of the publisher, the editors and the reviewers. Any product that may be evaluated in this article, or claim that may be made by its manufacturer, is not guaranteed or endorsed by the publisher.
